# Effect of Dicycloplatin, a Novel Platinum Chemotherapeutical Drug, on Inhibiting Cell Growth and Inducing Cell Apoptosis

**DOI:** 10.1371/journal.pone.0048994

**Published:** 2012-11-12

**Authors:** Guang-quan Li, Xing-gui Chen, Xing-ping Wu, Jing-dun Xie, Yong-ju Liang, Xiao-qin Zhao, Wei-qiang Chen, Li-wu Fu

**Affiliations:** 1 Department of General Surgery, Chen Xing Hai Hosital, Guangdong Medical College, Zhongshan, People’s Republic of China; 2 State Key Laboratory of Oncology in Southern China, Cancer Center, Sun Yat-Sen University, Guangzhou, People’s Republic of China; Complutense University, Spain

## Abstract

Dicycloplatin, a new supramolecular platinum-based antitumor drug, has been approved by the State Food and Administration (SFDA) of China. In this study, we investigated the anticancer activity of dicycloplatin in cancer cells and signaling pathways involved in dicycloplatin-induced apoptosis. Dicycloplatin inhibited the proliferation of cancer cells and increased the percentage of apoptosis in a concentration-dependent manner. Besides, some apoptosis related events were observed after treatment with dicycloplatin, including increase of reactive oxygen species (ROS), collapse of mitochondrial membrane potential (Δψm), release of cytochrome c from the mitochondria to the cytosol, upregulation of p53, which were accompanied by activation of caspase-9, caspase-3, caspase-8, and poly (ADP-ribose) polymerase cleavage in a concentration-dependent manner. The role of apoptosis in dicycloplatin-mediated cell death was further confirmed by the concomitant treatment with caspase-8 or caspase-9 inhibitors, which inhibited apoptosis and PARP cleavage. Intracellular glutathione (GSH) was also found to inhibit the cytotoxic effect of dicycloplatin. In conclusion, these findings suggest that dicycloplatin induces apoptosis through ROS stress-mediated death receptor pathway and mitochondrial pathway which is similar to carboplatin.

## Introduction

Platinum-based anticancer drugs, such as cisplatin and carboplatin, are the first-line clinical anticancer drugs for the treatment of solid tumors [1], including lung, breast, ovarian and head and neck cancers. Both cisplatin [2], [3], [4], [5] and carboplatin [6], [7], [8], [9] have been shown to inhibit cancer cells growth and induce apoptosis in cancer cells through death receptor pathway and/or mitochondrial pathway. However, the application of these drugs in the clinic is limited by their severe toxicities, such as nephrotoxicity, neurotoxicity and ototoxicity with cisplatin [10], [11] and myelosuppression with carboplatin [11]. Therefore, development of novel platinum anticancer drugs with high efficiency and low toxicity is warranted for cancer chemotherapy.

Dicycloplatin is a novel platinum-based anticancer drug which was formed by one molecule of carboplatin and one molecule of 1,1-ring succinic acid interacting intra-molecularly via hydrogen bond. It was shown to be highly soluble and stable, comparable with carboplatin [12]. While the anticancer efficacy of dicycloplatin was found to be more superior than cisplatin, the former has only minimal toxicity [13]. Animal studies also showed that dicycloplatin is much less nephrotoxic than cisplatin and it has similar myelosuppressiove effect as carboplatin [14]. It has been reported recently that dicycloplatin could inhibit the proliferation of a variety of tumor cells *in vitro* and *in vitro* via the induction of apoptosis [15], [16], [17]. More importantly, data from clinical trials indicated that the combination of dicycloplatin with paclitaxel in advanced non-small cell lung cancer was safe and effective, which further advocating the clinical development of dicycloplatin as a novel anticancer drug [18]. However, the mechanism by which dicycloplatin induced apoptosis in cancer cells remained unclear.

Apoptosis controls the development and homeostasis of multi-cellular organisms through a highly supervised and organized death process. The death receptor pathway and the mitochondrial pathway are the two major routes of apoptosis [19]. In the mitochondrial pathway, dysfunction of mitochondria induces a cascade of events, including release of cytochrome c from mitochondria to cytosol, binding of cytochrome c to apoptotic protease activating factor 1 (Apaf-1), sequential activation of procaspase-9 and caspase-3 and eventually leading to apoptosis [20]. Increasing evidences indicated that the Bcl-2 family proteins played an important role in regulating the mitochondrial-dependent cell apoptosis [21]. Bcl-2 family proteins comprise anti-apoptotic subfamily and pro-apoptotic subfamily. Bcl-2 and Bcl-xl belong to the anti-apoptotic, whereas Bax, Bak as well as Bid and Bad belong to the pro-apoptotic subfamily [22]. During the apoptosis process, pro-apoptotic subfamily member proteins, such as Bax, Bad and Bid, transmit to the mitochondria, and promote the release of cytochrome c [21]. The increased cytochrome c activates caspases and induces apoptosis. Additionally, intracellular reactive oxygen species (ROS) could also influence the mitochondrial pathway [23], [24], [25]. Overproduction of ROS could change the inner membrane permeability and inner membrane potential (Δψm) and induce release of cytochrome c from mitochondria to cytosol, finally resulting in apoptosis [26], [27]. Fas is the most typical death receptor in the death receptor pathway. After binding with its ligand FasL, Fas is activated and trimerizated to recruit the Fas-associated death domain (FADD) and procaspase-8, and finally forming the death-inducing signaling complex (DISC) [28]. Procaspase-8 is activated by autocatalysis, and the activated caspase-8 stimulates apoptosis directly via directly activates caspase-3 [29] or cleaves Bid into truncated Bid (tBid) which transmits to mitochondria and promotes the release of cytochrome c from mitochondria to the cytosol [30].

**Figure 1:**
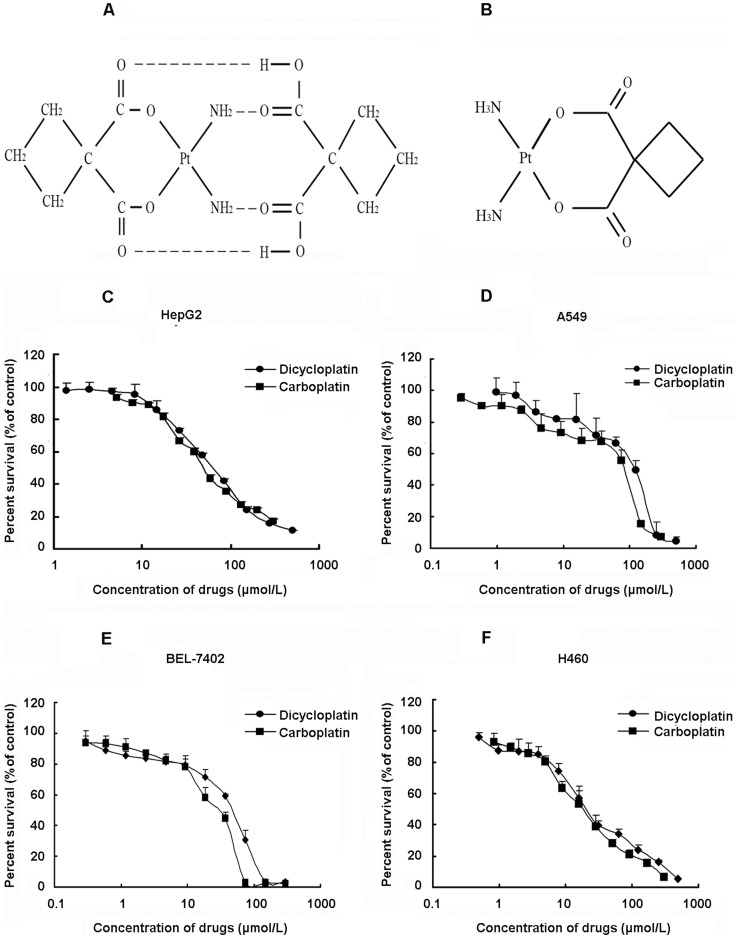
Cytotoxic effect of dicycloplatin in cancer cell lines. A, structure of dicycloplatin. B, structure of carboplatin. C–F, cytotoxicity of dicycloplatin and carboplatin in HepG2, A549, BEL-7402 and H460 cells. Cytotoxicity was measured by MTT assay. The cells were exposed to a full range of concentrations of dicycloplatin and carboplatin for 72 h. Cell viability with a model 550 microplate reader after staining with MTT for 4 h. The data presented represent mean±SD of three independent experiments.

In our study, dicycloplatin demonstrated superior potency in inhibiting growth and inducing apoptosis than the classical platinum-based anticancer drugs in cancer cells. The detailed mechanism leading to dicycloplatin-induced apoptosis was investigated and compared with that of carboplatin.

**Figure 2:**
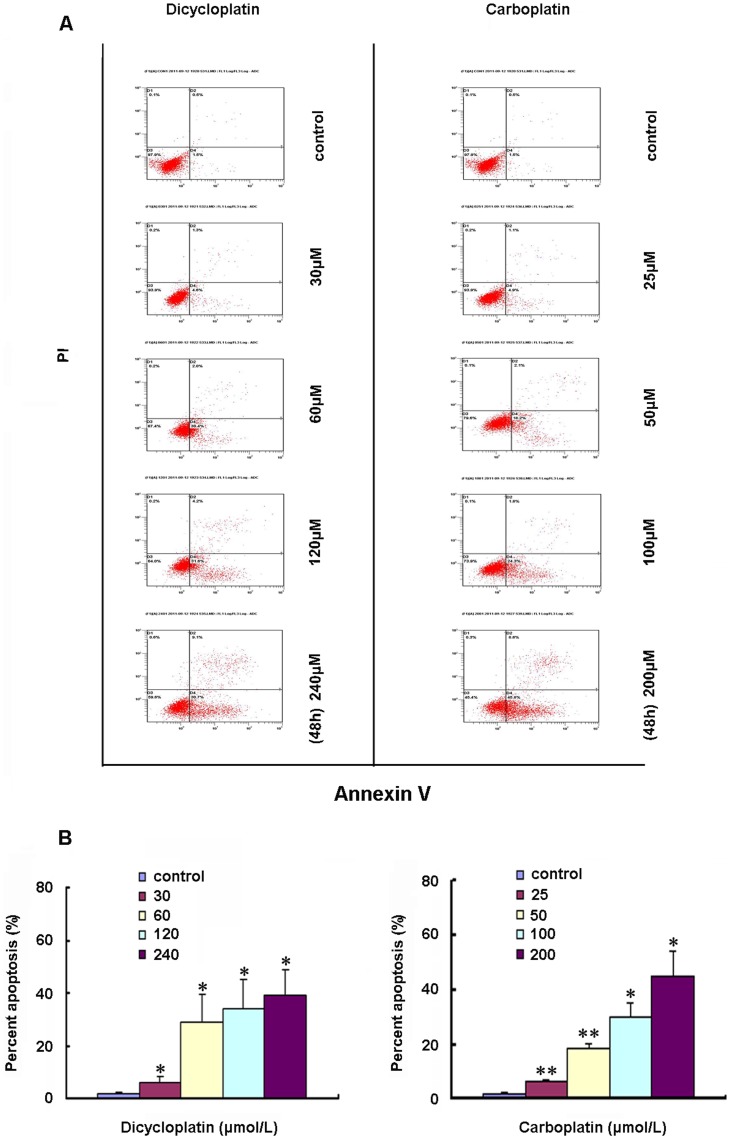
Induction of apoptosis by dicycloplatin in HepG2 cells. A, apoptosis analysis in HepG2 cells was assessed by Annexin V/PI double staining. After cells were exposed to the three designated concentrations of dicycloplatin or carboplatin for 48 h, respectively, the attached and detached cells were collected. Following staining with Annexin V and PI, cells were subjected to flow cytometry analysis. Bottom right quadrant, cells stained mainly by Annexin V (early apoptotic cells); top right quadrant, cells stained by both PI and Annexin V (late apopototic/necrotic secondary necrosis); top left quadrant, cells stained mainly by PI viable cells; bottom left quadrant, cells negative for both Annexin V and PI. B, percent apoptosis. Early apoptotic cell population with Annexin V-positive but PI-negative cells increased gradually from 1.5% to 31.6% and 1.5% to 45% in dicycloplatin- and carboplatin-treated HepG2 cells, respectively. The data presented represents the mean±SD from three independent experiments (*, *P*<0.05; **, *P*<0.01).

**Figure 3:**
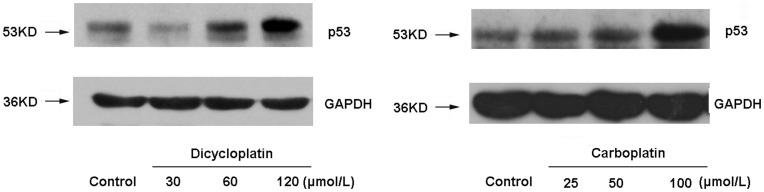
Up-regulation of p53 expression by dicycloplatin in HepG2 cells. After HepG2 cells were treated with the three designated concentrations of dicycloplatin (30, 60 or 120 µmol/L) and carboplatin (25, 50 or 100 µmol/L) for 48 h, respectively, the mitochondria and cytosolic fractions or the whole-cell lysates were assayed by Western blot analysis and corresponding antibodies. Glyceraldehyde-3-phosphate dehydrogenase detection was used to acertain equal protein loading.

**Figure 4:**
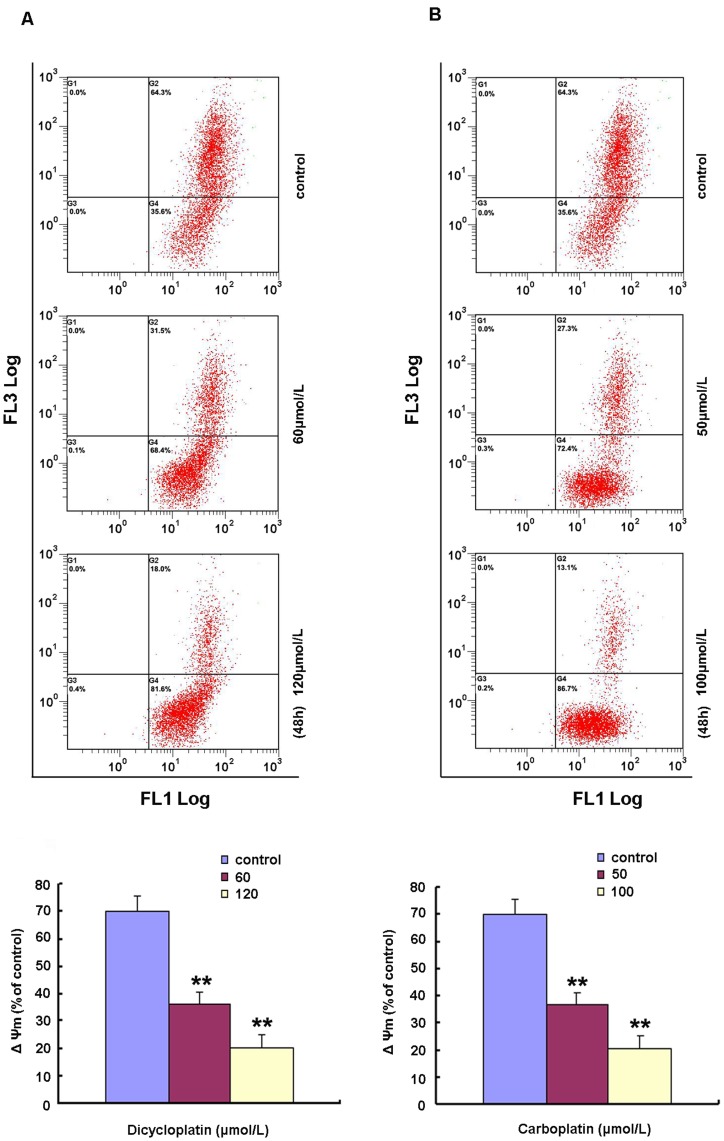
Induction of Δ**Ψm collapse by dicycloplatin in HepG2 cells.** A and B, dicycloplatin and carboplatin treatment resulted in the loss of ΔΨm. HepG2 cells was exposed to 60 or 120 µmol/L dicycloplatin and to 50 and 100 µmol/L carboplatin for 48 h, respectively. The ΔΨm was determined by flow cytometry. Top, the ΔΨm in both dicycloplatin- and carboplatin-treated cells decreased in a concentration-dependent manner. Loss of ΔΨm was measured using JC-1 staining. JC-1 was a cell-penetrating lipophilic cationic fluorochrome. Cells containing forming J-aggregates have high ΔΨm, and show red fluorescence (FL3). Cells with low ΔΨm are those in which JC-1 maintains (or reacquires) monomeric form, and show green fluorescence (FL1). Depolarization of ΔΨm was measured by JC-1, which accumulates in mitochondrial matrix, driven by ΔΨm, and expressed as an increase of green to red fluorescence ratio reflecting the transformation of JC-1 aggregates into monomers when mitochondrial membrane becomes depolarized. Bottom, ΔΨm levels of dicycloplatin- and carboplatin-treated HepG2 cells, expressed as units of mean fluorescence intensity, were calculated as percentage of control. Data are mean±SD of three independent experiments (**, *P*<0.01).

**Figure 5:**
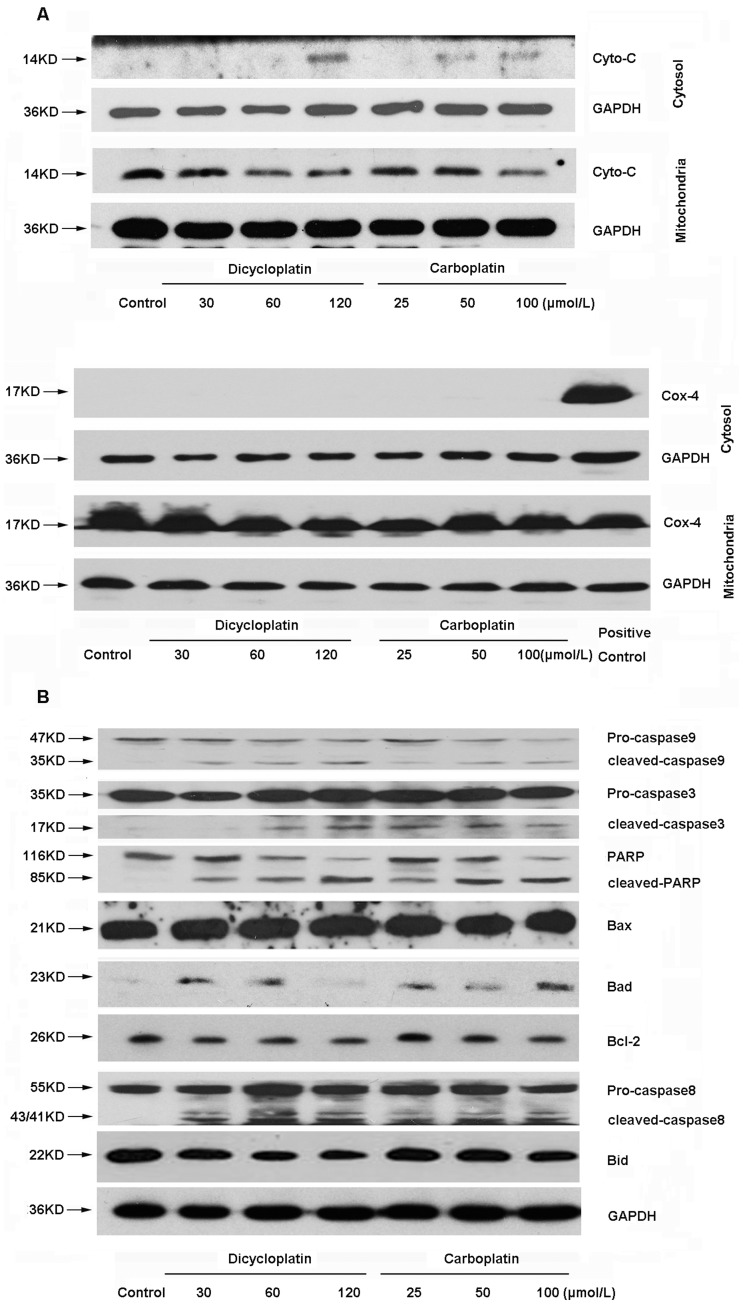
Involvement of mitochondrial pathway and death receptor pathway in dicycloplatin-induced apoptosis in HepG2 cells. After HepG2 cells were treated with 30 to 120 µmol/L dicycloplatin or 25 to 100 µmol/L carboplatin for 48 h, respectively, the mitochondria and cytosolic fractions or the whole-cell lysates were assayed by Western blot analysis with the corresponding antibodies. A, mitochondrial cytochrome c release into cytosol and the cytochrome c in mitochondria. Cox-4 detection was used to confirm that the complete separation of cytochrome c in the mitochondrial and cytosolic fractions. B, activations of caspase-9 and caspase-3 and the cleavage of PARP in dose-dependent manner were observed. In addition, both dicycloplatin and carboplatin could down-regulate the expression of Bcl-2 and up-regulate that of Bad but did not influence the level of Bax. Moreover, activations of caspase-8 and decrease of native Bid were also observed.

**Figure 6:**
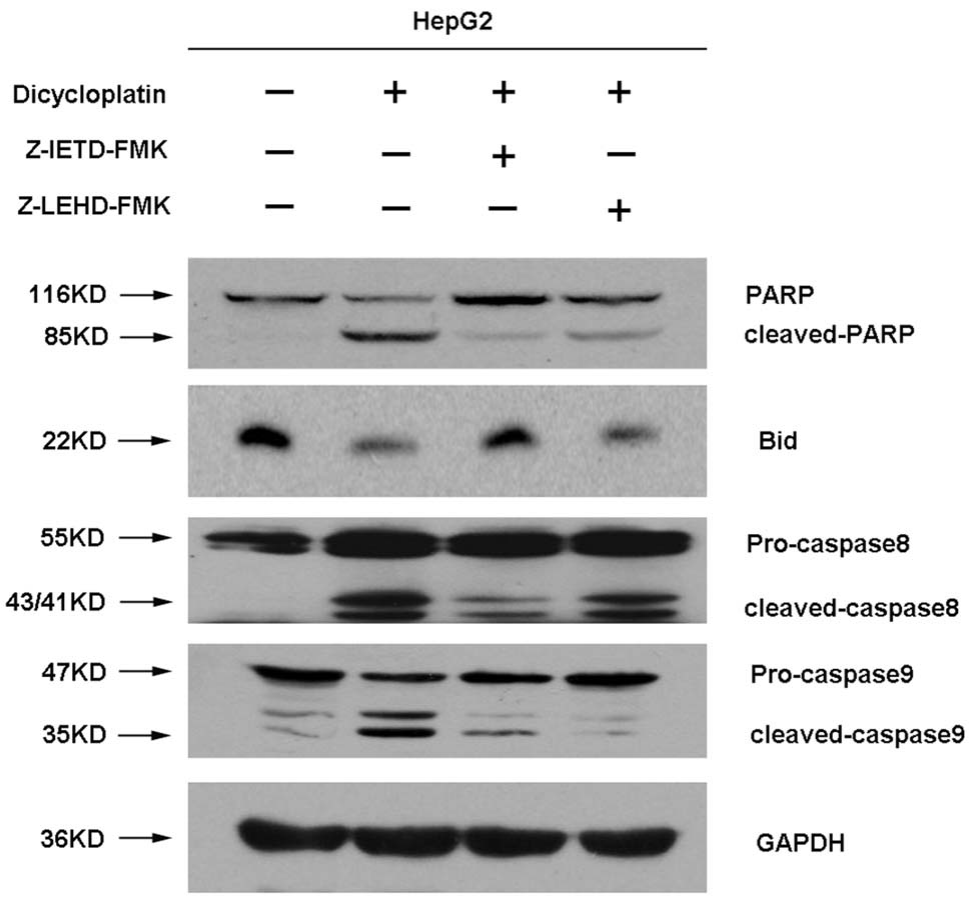
Impairment of dicycloplatin-induced PARP cleavage by caspase 8 and caspase 3 inhibition. HepG2 cells were treated with dicycloplatin alone or pretreated with Z-IETD-FMK (60 µmol/L) or Z-LETD-FMK (60 µmol/L) for 24 h and then incubated with dicycloplatin (60 µmol/L) for 48 h. Glyceraldehyde-3-phosphate dehydrogenase detection was used to confirm equal protein loading. Both Z-IETD-FMK and Z-LETD-FMK could reduce PARP cleavage, whereas Bid cleavage was inhibited by Z-IETD-FMK only.

**Figure 7:**
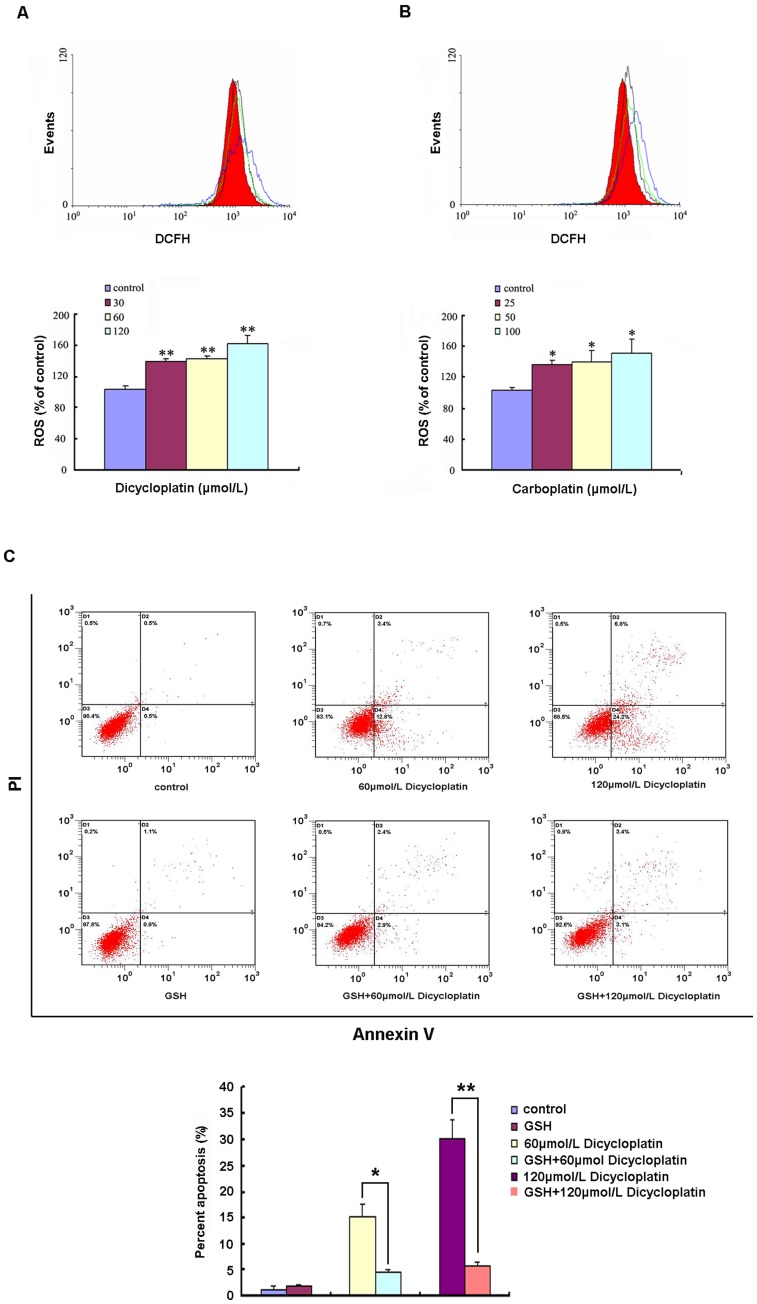
Induction of ROS was involved in dicycloplatin-induced apoptosis in HepG2 cells. A and B, dicycloplatin and carboplatin induced increase of ROS generation in HepG2 cells. After HepG2 cells were exposed to 30 to 120 µmol/L dicycloplatin or 25 to 100 µmol/L carboplatin for 48 h, respectively, intracellular ROS levels were analyzed by flow cytometry. Significant increases of ROS production were observed in HepG2 cells after dicycloplatin and carboplatin treatment. Top, ROS levels in dicycloplatin and carboplatin-treated cells, respectively. Red area and black, green, and blue lines, ROS levels for untreated control and 30, 60, 120 µmol/L dicycloplatin treatment groups, or for untreated control and 25, 50, 100 µmol/L carboplatin treatment groups, respectively. Bottom, ROS levels of dicycloplatin- and carboplatin-treated cells, expressed as units of mean fluorescence intensity, were calculated as percentage of control. The data presented represents mean±SD from three independent experiments (*, *P*<0.05; **, *P*<0.01). C, GSH significantly inhibited apoptosis induced by dicycloplatin. HepG2 cells were pretreated with GSH (5 mmol/L) for 1 h and then incubated with dicycloplatin (60, 120 µmol/L) for 48 h, respectively. The attached and detached cells were collected. Following staining with Annexin V and PI, cells were subjected to flow cytometer analysis. Bottom right quadrant, cells stained mainly by Annexin V (early apoptotic cells); top right quadrant, cells stained by both PI and Annexin V (late apopototic/necrotic secondary necrosis); top left quadrant, cells stained mainly by PI viable cells; bottom left quadrant, cells negative for both Annexin V and PI. Bottom, percent apoptosis. Percent of apoptosis induced by dicycloplatin was significantly inhibited by GSH. Each data represents the mean±SD from three independent experiments (*, *P*<0.05; **, *P*<0.01).

**Figure 8:**
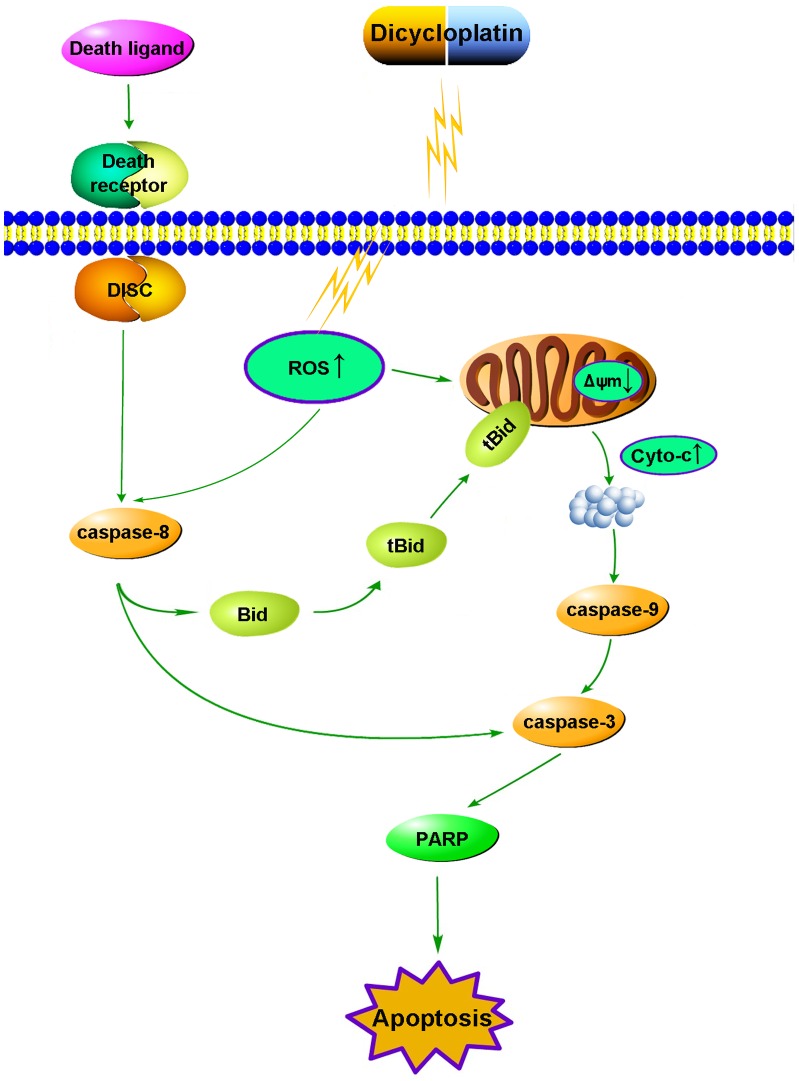
Proposed apoptotic pathway induced by dicycloplatin.

## Materials and Methods

### Chemicals and Reagents

3-(4,5-Dimethylthiazol-2-yl)-2,5-diphenyltetrazolium bromide (MTT), and 2′,7′-dichlorofluorescin diacetate were purchased from Sigma. 5,5′,6′6′-tetrachloro-1,1′,3,3′-tetraethylbenzimidazolcarbocyanine iodide (JC-1) and glutathione (GSH) were purchased from Beyotime Institute. Adriamycin was purchased from ZhuHai MingZHi Pharmaceuticals. ApopNexin FITC Apoptosis Detection Kit was purchased from Chemicon. Caspase-9 inhibitor (Z-LETD-FMK) and caspase-8 inhibitor (Z-IETD-FMK) were purchased from Biovision. Antibodies against caspase-3, cytochrome c and p53 were obtained from Santa Cruz Biotechnology. Antibodies against caspase-8, caspase-9, poly (ADP-ribose) polymerase (PARP), Bad, Bax, Bid and Cox4 were obtained from Cell Signaling Technology. Antibodies against glyceraldehydes-3-phosphate dehydrogenase, anti-mouse IgG-horseradish peroxidase, and anti-rabbit IgG-horseradish peroxidase were purchased from KangCheng Biotechnology. All tissue culture supplies were purchased from Life Technologies. Other routine laboratory reagents were obtained from Whiga Biotechnology of analytical or high-performance liquid chromatography grade. Supramolecular complexes dicycloplatin was obtained from Beijing Xing DA Science Systems, Inc (Figure 1A).

### Cell Lines and Cell Culture

The following cell lines were cultured in DMEM or RPMI 1640 supplemented with 10% FBS at 37°C in a humidified atmosphere of 5% CO2, respectively, which contain 100 units/mL penicillin, 100 µg/mL streptomycin, and 10% fetal bovine serum. The human hepatoma cell lines HepG2 and BEL-7402 were obtained from School of Biomedical Sciences, The Chinese University of HongKong [31] and Institute of Medicinal Biotechnology, Chinese Academy of Medical Science and Peking Union Medical College [32], respectively. The human lung cancer cell lines A549 and H460 were obtained from Cancer Hospital Chinese Academy of Medical Science [33] and GuangZhou Institute of Respiratory Disease [34], respectively.

### Cell Viability Assay

MTT assay measures the activity of mitochondrial dehydrogenase enzymes basing on its ability of cleaving tetrazolium ring to produce formazan; therefore, the assay can be used as an index of cell viability. Cells were harvested during logarithmic growth phase and seeded in 96-well plates at a density of 3.5×104/mL in a final volume of 190 µL/well. After 24 h incubation, 10 µL dicycloplatin full-range concentration was added to 96-well plates. After 68 h treatment, 20 µL MTT (20 mg/mL stock solution of saline) was added to each well for 4 h. Subsequently, the supernatant was removed, and MTT crystals were solubilized with 200 µL anhydrous DMSO in each well. Thereafter, cell viability was measured with a model 550 microplate reader (Bio-Rad) at 540 nm, with 655 nm as reference filter [35]. The 50% inhibitory concentration (IC50) was determined as the anticancer drug concentration causing 50% reduction in cell viability and calculated from the cytotoxicity curves (Bliss’ software). Cell percent survival was calculated using the following formula: survival (%)  =  [(mean experimental absorbance)/(mean control absorbance)]×100%.

### Annexin V/Propidium Iodide Double-Staining Assay

Annexin V and propidium iodide (PI) staining was done using ApopNexin FITC Apoptosis Detection Kit. Cells (6×105) were seeded in 25 cm^2^ flasks and allowed to attach for 24 h. After a 48-h treatment with the desired concentration dicycloplatin, both floating and attached cells were collected, washed with ice-cold PBS twice, and resuspended in 200 µL 1×binding buffer containing Annexin V (1∶50 according to the manufacturer’s instruction) and 40 ng/sample PI for 15 min at 37°C in the dark [36]. Then, the number of viable, apoptotic, and necrotic cells was quantified by flow cytometer (Becton Dickinson) and analyzed by the Cell-Quest software. Cells were excited at 488 nm and the emissions of Annexin V at 525 nm and PI were collected through 610 nm band-pass filters. At least 10,000 cells were analyzed for each sample. Percent apoptosis (%)  =  [(number of apoptotic cells)/(number of total cells observed)]×100%.

### Determination of Mitochondrial Membrane Potential

Mitochondrial membrane potential (ΔΨm) was measured by flow cytometry with the mitochondrial tracking fluorescent dye 5,5′,6′6′-tetrachloro-1,1′,3,3′-tetraethylbenzimidazolcarbocyanine iodide (JC-1). JC-1 was a cell-penetrating lipophilic cationic fluorochrome. Cells containing forming J-aggregates have high ΔΨm, and show red fluorescence (FL3). Cells with low ΔΨm are those in which JC-1 maintains (or reacquires) monomeric form, and show green fluorescence (FL1). Depolarization of ΔΨm was measured by JC-1, which accumulates in mitochondrial matrix, driven by ΔΨm, and expressed as an increase of green to red fluorescence ratio reflecting the transformation of JC-1 aggregates into monomers when mitochondrial membrane becomes depolarized. After HepG2 cell was exposed to 60 to120 µmol/L dicycloplatin for 48 h, 6×10^5^ cells were harvested, centrifuged at 1,000 rpm for 5 min, and washed with ice-cold PBS once. Thereafter, cells were incubated with JC-1 at 37°C for 20 min. At least 10,000 cells were determined for each sample. The data obtained from flow cytometry were analyzed by CellQuest software. A representive result from at least three independent experiments is presented.

### Measurement of Reactive Oxygen Species Generation

2′,7′-Dichlorofluorescin diacetate is a cell permeable fluorescent tracer specific for reactive oxygen species (ROS) assessment. It can be deacetylated by intracellular esterase to the nonfluorescent 2′,7′-dichlorofluorescin, which is oxidized by ROS to the fluorescent compound 2′,7′-dichloroflorescein. Thus, the fluorescence intensity of 2′,7′-dichloroflorescein is proportional to the amount of ROS produced by the cells. After HepG2 cells were exposed to 30 to 120 µmol/L dicycloplatin for 48 h, 6×10^5^ cells were harvested, washed once with ice-cold PBS, and incubated with 50 µmol/L 2′,7′-dichloroflorescein diacetate at 37°C for 20 min in the dark. Then, the cells were washed twice and maintained in 1 mL PBS. The ROS generation was assessed from 10,000 cells each sample by FACS Calibur flow cytometer at the excitation wavelength of 488 nm and emission wavelength of 530 nm [37]. The data of 2′,7′-dichloroflorescein fluorescence intensity were evaluated by CellQuest software and expressed as mean fluorescence intensity. The assays were repeated at least three times.

### Whole-Cell Lysates and Western Blot Analysis

After HepG2 cells were exposed to 30 to 120 µmol/L dicycloplatin for 48 h, respectively [preincubated with Z-IETD-FMK (a caspase-8 inhibitor) or Z-LETD-FMK (a caspase-9 inhibitor)], whole cells were harvested and washed twice with ice-cold PBS, and the pellet was vortexed and 1×lysis buffer [50 mmol/L Tris-HCl (pH6.8), 10% glycerol, 2% SDS, 0.25‰ bromophenol blue, and 0.1 mol/L DTT ] was added for 100 µL/5×106 cells. After heated at 95°C for 20 min, the lysates were centrifuged at 12,000 rpm for 10 min and the supernatant was collected [38]. The protein concentration was determined by nucleic acid-protein analyzer (Beckman). Equal amount of lysate protein was separated on 8% to 12% SDS-PAGE and transferred onto polyvinylidene difluoride membrane (Pall). The nonspecific binding sites were blocked with TBST buffer [150 mmol/L NaCl, 20 mmol/L Tris-HCl (pH7.4), and 0.4% (v/v) Tween 20] containing 5% nonfat dry milk for 2 h. The membranes were incubated overnight at 4°C with specific primary antibodies. Then, the membranes were washed three times with TBST buffer and incubated at room temperature for 1 h with horseradish peroxidase-conjugated secondary antibody. After washed thrice with TBST buffer, the immunoblots were visualized by the enhanced Phototope-Horseradish Peroxidase Detection Kit purchased from Cell Signaling Technology and exposed to Kodak medical X-ray processor [37].

### Subcellular Fractionation for Western Blot Analysis of Cytosolic Cytochrome c

After HepG2 cells were exposed to 30 to 120 µmol/L dicycloplatin for 48 h, whole cells were harvested by centrifugation at 1,000 rpm for 5 min. The pellets were washed twice with ice-cold PBS, suspended with 5-fold volume ice-cold cell extract buffer [20 mmol/L 4-(2-hydroxyethy1)-1-piperazineethanesulfonic acid (HEPES-KOH; pH7.5), 10 mmol/L KCl, 1.5 mmol/L MgCl2, 1 mmol/L EDTA, 1 mmol/L EGTA, 1 mmol/L DTT, 250 mmol/L sucrose, 0.1 mmol/L phenylmethylsulfonyl fluoride, and 0.02 mmol/L aprotinin], and incubated for 40 min at 4°C. Then, the cells were centrifuged at 1,200 rpm for 10 min at 4°C and the final supernatant was used as cytosolic fraction of cytochrome c. Then, 5×loading buffer [250 mmol/L Tris-HCl (pH6.8), 50% (v/v) glycerol, 10% (w/v) SDS, 0.5% (w/v) bromphenol blue, and 5% (w/v) DTT] was added to the above obtained supernatant and the mixture was boiled at 100°C for 15 min. Thus, the protein solution was used for identification of cytosolic cytochrome c by Western blot with 15% SDS-PAGE and blotting onto polyvinylidene difluoride membrane. The cytochrome c protein was detected by using anti-cytochrome c antibody in the ratio of 1∶1,000 [39].

### Statistical Analysis

Results were done by t-test or one-way ANOVA with SPSS 13.0 software. Data was presented as mean±SD of at least triplicate determinations. *, *P*<0.05 was indicative of significant difference and **, *P*<0.01 was indicative of very significant difference.

## Results

### Dicycloplatin Inhibited Proliferation in a Variety of Tumor Cells

The inhibition of cancer cell proliferation by dicycloplatin was examined by MTT assay. As shown in Figure 1, dicycloplatin inhibited cell proliferation in a concentration-dependent manner, and the IC50 of dicycloplatin was 61.30±6.33 µmol/L, 89.80±6.14 µmol/L, 41.69±4.32 µmol/L and 20.25±3.43 µmol/L in HepG2, A549, BEL-7402 and H460 cells, respectively. Comparable IC50 values were also obtained for carboplatin in these cancer cell lines (Figure 1): Carboplatin’s IC50 was 48.01±2.45 µmol/L, 83.20±2.38 µmol/L, 30.27±3.18 µmol/L and 20.44±1.98 µmol/L, respectively.

### Induction of Apoptosis by Dicycloplatin

To assess apoptosis induced by dicycloplatin in cancer cells, we detected the exposure of phosphatidylserine on the cell surface, which is known to be an early event in the initiation of apoptosis, by using ApopNexin FITC Apoptosis Detection Kit. As shown in Figure 2A, the apoptotic cell population increased gradually with concentration in dicycloplatin-treated (30, 60, 120 and 240 µmol/L) HepG2 cells. The percentage of early apoptosis in the untreated cell was 1.73±0.4%, which was increased to 6.20±1.8%, 28.83±1.1%, 33.87±1.1% and 39.00±9.8% after a 48-h treatment with dicycloplatin at 30, 60, 120 and 240 µmol/L for 48 h, respectively (Figure 2B). Similar results were observed in carboplatin-treated HepG2 cells over a range of different concentrations (25, 50, 100 and 200 µmol/L) (Figure 2).

### Dicycloplatin Upregulated the Expression of p53

Dicycloplatin is a novel supramolecular platinum chemotherapeutical drug which was formed by one molecule of carboplatin and one molecule of 1,1-ring succinic acid interacting intramolecularly via hydrogen bonding. Since platinum-based anticancer drugs, such as cisplatin and carboplatin, are to DNA-damaging agents, they were known to induce apoptosis partially by affecting p53 expression in cancer cells. As shown in Figure 3, dicycloplatin, like carboplatin, also upregulated the expression of p53 in a concentration-dependent manner.

### Change of Mitochondria Membrane Potential (ΔΨm) Induced by Dicycloplatin

Imbalance of mitochondrial membrane potential (ΔΨm) is a commonly reported phenomenon of the early stage of the apoptosis. To explore whether dicycloplatin changed ΔΨm, JC−1 was used for its detection. As shown in Figure 4A, a decline of ΔΨm was observed in dicycloplatin-treated HepG2 cells in a concentration-dependent manner. ΔΨm was decreased from 70.0±5.51% in untreated cells to 36.4±4.6% and 20.2±4.9% after treatment with 60 µmol/L and 120 µmol/L of dicycloplatin, respectively. As shown in Figure 4B, similar results were obtained for carboplatin in HepG2 cells.

### Apoptosis Induced by Dicycloplatin was Dependent on Death Receptor Pathway and Mitochondrial Pathway

Mitochondrial pathway is one of the major apoptosis pathways, in which cytochrome c is the limiting factor. Mitochondrial dysfunction has been suggested to cause the release of cytochrome c. Moreover, increasing evidence suggested that Bcl-2 protein family forms a link between death receptor pathway and mitochondrial apoptotic pathway [40]. The levels of cytochrome c in mitochondria and cytoplasm, caspases, and other apoptotic related proteins were examined by Western blot analysis in HepG2 cells after treatment with different concentration of dicycloplatin (30, 60 and 120 µmol/L). As shown in Figure 5A, cytochrome c was decreased in mitochondrial and increased in cytosolic fraction in HepG2 cells after dicycloplatin treatmented. Furthermore, as shown in Figure 5B, the activation of caspase-9 and caspase-3 and the cleavage of PARP were also observed in a dose-dependent manner. It is noteworthy that upregulation of Bad and downregulation of Bcl-2 were observed, but Bax expression was not altered. Additionlly, activation of caspase-8 and cleavage of Bid (decrease of native Bid) were also observed. These data implied that both mitochondrial pathway and death receptor pathway were involved in the apoptotic process induced by dicycloplatin in HepG2 cells, which is similar to carboplatin (Figure 5).

### Cleavage of Bid Induced by Dicycloplatin were Inhibited by Caspase-8 Inhibitor

It is increasingly believed that the cleavage form of Bid (tBid) caused by caspase-8 can result in the release of cytochrome c from mitochondria into cytosol, and subsequently activating caspase-9. Caspase-8 inhibition study was thus carried out to clarify whether the activation of caspase-9 induced by dicycloplatin is the downstream event of caspase-8 activation. A caspase-8 inhibitor (Z-IETD-FMK, 60 µmol/L) was used to pretreat HepG2 cells for 24 h, and then the cells were treated with 60 µmol/L dicycloplatin for 48 h. Whole-cell lysates were examined by Western blot analysis for the status of the caspase-9 and caspase-8 cascade with the corresponding antibodies. As shown in Figure 6, suppression of caspase-8 activity with Z-IETD-FMK was found to inhibit cleavage of Bid and PARP induced by dicycloplatin. On the other hand, a much less significant inhibition of Bid and PARP cleavage after dicycloplatin was observed when caspase-9 was suppressed by Z-LETD-FMK.

### Apoptosis Induced by Dicycloplatin was Dependent on ROS

It is known that ROS plays a certain role in apoptosis induced by some anticancer drugs. In our studies, excessive generation of ROS was observed in dicycloplatin- and carboplatin-treated HepG2 cells (Figure 7A & B). To explore whether apoptosis induced by dicycloplatin was dependent on ROS, GSH (an anti-oxidants, 5 mmol/L) was used to pretreat HepG2 cells for 1 h, before the subsequent treatment with dicycloplatin at different concentrations (60 to 120 µmol/L) for 48 h. As shown in Figure 7C, the percentage of apoptosis induced by dicycloplatin was remarkably inhibited by GSH. These results indicated that apoptosis induced by dicycloplatin in HepG2 cells was likely dependent on ROS.

## Discussion

Cisplatin and carboplatin, the classical platinum-based antitumor drugs, are commonly used as the first-line treatment for a number of solid malignancies. However, the clinical application of these drugs is limited by their severe toxicity. Dicycloplatin, a novel platinum chemotherapeutical drug has been reported to exhibit lower toxicity and better anticancer activity compared with cisplatin and carboplatin [13], [14], [18]. Dicycloplatin is a promising anticancer agent and it has been approved by the SFDA of China. At present, the anticancer mechanism of dicycloplatin is still unclear. This study was aimed to understand the mechanism of action of dicycloplatin and to compare and contrast its mechanism of causing apoptosis versus that of carboplatin.

Our result indicated that dicycloplatin and carboplatin exhibited similar anti-proliferation effect in HepG2 cells with IC50 of 61.30±6.33 µmol/L and 48.01±2.45 µmol/L, respectively (Figure 1C). Moreover, Annexin V/PI double staining assay confirmed that dicycloplatin and carboplatin induced apoptosis in HepG2 cells in a concentration-dependent manner (Figure 2). Taken together, these results indicated that dicycloplatin, like carboplatin, inhibited the proliferation of HepG2 cells via inducing cell apoptosis.

Mitochondrial dysfunctions, such as loss of ΔΨm, permeability transition, and release of cytochrome c from mitochondria into cytosol are the major causes of apoptosis [41]. In our study, decline of ΔΨm (Figure 4A & B) was detected in dicycloplatin- and carboplatin-treated HepG2 cells. Moreover, excessive emancipation of cytochrome c from mitochondria to cytosol (Figure 5A) and a decrease of cytochrome c in the mitochondria were also observed (Figure 5A). Furthermore, the activation of caspase-9, caspase-3, and the cleavage of PARP were also detected by Western blot analysis (Figure 5B). Bcl-2 family protein plays an important role in regulating the mitochondrial-dependent cells apoptosis [21]. It has been demonstrated that cisplatin and carboplatin could change the expressions of Bcl-2 family proteins when inducing apoptosis [9], [42], [43]. To this end, the increase of Bad and decrease of Bcl-2 were detected in dicycloplatin-mediated apoptosis (Figure 5B). These results indicated that mitochondrial pathway played a role in apoptosis process induced by dicycloplatin and carboplatin in HepG2 cells.

Fas is the most typical death receptor in death receptor pathway, whose binding with FasL can lead to caspase-8 activation. Activated caspase-8 can stimulate apoptosis by directly activating downstream caspase-3 [29] and/or by cleaving Bid [30]. Once Bid is cleaved by caspase-8, the cleavage of Bid (tBid) translocates to the mitochondria from the cytosol and cause mitochondrial dysfunction then promotes the release of cytochrome c from mitochondria to cytosol. Therefore, it is increasingly believed that Bid represents an important biochemical signal bridging the mitochondrial pathways and the death receptor pathways [44]. In our study, an increase of caspase-8 activation and cleavage of Bid (decrease of native Bid protein) were observed after dicycloplatin treatment (Figrue 5B). Furthermore, caspase-8 inhibitor Z-IETD-FMK obviously decreased tBid formation whereas this effect by the caspase-9 inhibitor Z-LETD-FMK was not so obvious compared with Z-IETD-FMK (Figure 6). We also found that both Z-IETD-FMK and Z-LETD-FMK could reduce PARP cleavage(Figure 6). These results suggested that death receptor pathway may be another possible mechanism involved in the apoptosis induced by dicycloplatin partly through tBid. Taken together, our results showed that both mitochondrial pathway and death receptor pathway took part in dicycloplatin- and carboplatin-mediated apoptosis in HepG2 cells (Figure 8).

Recent studies have demonstrated that generation of ROS and the disruption of redox homeostasis played a role in apoptosis which induced by some anticancer agents [45]. Excessive generation of ROS has been reported to cause mitochondrial dysfunctions [46], [47]. In our study, excessive generations of ROS (Figure 7A & B) was detected in dicycloplatin- and carboplatin-treated HepG2 cells. Furthermore, when HepG2 cells were pretreated with GSH (an anti-oxidant, 5 mmol/L), the percent of apoptosis induced by dicycloplatin was remarkably inhibited (Figure 7C). The result indicated that apoptosis induced by dicycloplatin was ROS dependent.

In conclusion, our data suggested that dicycloplatin could induce apoptosis in HepG2 cells via both mitochondrial pathway and death receptor pathway participated in apoptosis process. Moreover, these mechanisms were similar to carboplatin. Furthermore, the mitochondrial pathway was dependent on generation of ROS.
